# Do GenAI avatars open new responsibility gaps?

**DOI:** 10.1007/s00146-025-02660-9

**Published:** 2025-10-04

**Authors:** Mihaela Constantinescu

**Affiliations:** Research Center in Applied Ethics, Faculty of Philosophy, University of Bucharest, Bucharest, Romania

**Keywords:** Avatars, Responsibility gaps, Proxy relation, Generative artificial intelligence, Large language models, Robotics

## Abstract

In this article, I argue that semi-autonomous avatars relying on generative artificial intelligence to replicate or represent real human persons—GenAI avatars—open a new type of responsibility gaps, which I call “proxy gaps”. Proxy gaps refer to situations when we cannot hold anyone morally responsible for the outcomes of GenAI avatars, because the representation relationship between avatars and humans is shaped by multimodal Large Language Models (LLMs). In addition to epistemic gaps by AI avatars discussed in the literature, I argue that GenAI avatars also open control gaps—where no one really controls the output of the avatar. I introduce the “proxy-control paradox” to explain why control gaps arise: in trying to achieve improved control over the desired outcome of their avatar, humans need to delegate control over the process leading to that outcome to the GenAI technology. Together, the epistemic and control gaps complicate the two criteria traditionally used for moral responsibility, resulting in a proxy gap by GenAI avatars. Despite inherent proxy gaps, I argue that, under certain circumstances, we can still rightfully hold individuals morally responsible for the outcome of their GenAI avatars. I detail four conditions pertaining to human understanding and LLM personalization, as well as the right to veto and outcome control, which, taken together, can ground individual moral responsibility for the outcome of personal GenAI avatars.

## Introduction

Generative artificial intelligence powered avatars—GenAI avatars—that use multimodal Large Language Models (LLMs) to replicate or represent real human persons have recently enabled the production of high-quality outputs in digital environments including the Metaverse and Virtual Reality (VR), with use cases in economic, administrative, legal, and moral decision-making. These digital artificial replicas of human persons are fine-tuned to take on the behavior, gestures, speaking, writing, and visual appearance of real individuals. Importantly, generative AI technology facilitates bidirectional transfer between digital and physical domains, suggesting that our digital representations may soon be realized as actual robots. The next technological step might well involve transitioning our digital replicas into robotic embodiments and vice versa, with the body becoming an interface (Boddington 2021).

While digital GenAI avatars are currently highly autonomous and can perform tasks in the manner of a particular person without necessitating persistent and direct human control (Sweeney 2025), robotic GenAI avatars require higher degrees of teleoperation through VR headsets for optimal functionality, as an intermediate step towards more autonomous functioning (Dafarra et al. 2024; Ishiguro et al 2025). Despite varying degrees of autonomy, digital and robotic GenAI avatars still need human intervention, rendering them as rather semi-autonomous personal replicas: robotic avatars necessitate a human operator most of the time, while digital GenAI avatars necessitate one from time to time.

Widespread use of GenAI avatars introduces novel ethical challenges for attributions of responsibility, notably in terms of responsibility gaps—indeterminacy with respect to whom can be held morally responsible for their outputs. GenAI avatars bring a fundamental shift in how artificial entities interact with and represent humans in digital and physical spaces. Using transformer architectures, these avatars display human-level linguistic competencies, successfully passing numerous benchmark tests. They can largely learn independently, and can self-prompt, thus demonstrating greater autonomy compared to previous AI systems. Unlike other AI systems designed for specific functional purposes, these avatars possess general capabilities and are designed to embody the identity, personality, and characteristics of specific individuals; unlike prior use of avatars that did not integrate generative AI, current use of LLMs actively shapes the relationship between the avatar and the person they instantiate. This raises relevant questions about representation, control, and attribution of moral responsibility. As a result, GenAI avatars might open new responsibility gaps adding to the ones already discussed in relation to highly autonomous AI systems (for an overview see Nyholm 2023, ch. 6; Oimann and Tollon 2024).

In this article, I argue that GenAI avatars lead to a new type of responsibility gaps, which I call “proxy gaps”: situations when we cannot hold anyone morally responsible for the outcomes of GenAI avatars, given the representation relationship between avatars and their human counterparts, shaped by LLMs. Paula Sweeney (2023) has argued that AI-powered avatars understood as proxies, i.e., avatars that both represent and replace individual human persons, open epistemic gaps—where no one really knows if, faced with a new context, the avatar will make decisions as the represented human would. In addition, I argue that GenAI avatars also open control gaps—where no one really controls the output of the avatar. I introduce the “proxy-control paradox” to explain why control gaps arise: in trying to achieve improved control over the desired outcome of their avatar, humans need to delegate control over the process leading to that outcome to the GenAI technology. Together, the epistemic and control gaps complicate the two criteria traditionally used for moral responsibility, resulting in a “proxy gap” opened by use of GenAI avatars.

Despite inherent proxy gaps, I argue that, under certain circumstances, we can still rightfully hold individuals morally responsible for the outcome of their GenAI avatars. I detail four conditions pertaining to human understanding and LLM personalization, as well as the right to veto and outcome control, which, taken together, can ground individual moral responsibility for the outcome of personal GenAI avatars.

I start by briefly explaining responsibility gaps opened by AI systems and highlighting complications brought by GenAI avatars (Sect. 2). I then introduce the idea of “proxy-control paradox” determined using this type of avatars (Sect. 3) and move on to develop the point that GenAI avatars open “proxy-responsibility gaps” (Sect. 4). In the fifth and last section, I explore a possible solution to proxy-responsibility gaps by highlighting the way individuals might bear moral responsibility for the actions of their avatars, provided several criteria are met. I end with the recommendation that the use of semi-autonomous avatars is preferable to fully autonomous avatars, given the criteria that bridge proxy gaps.

## Responsibility gaps, AI systems, and GenAI avatars

It is already two decades since AI ethics research has discussed problems of responsibility attributions for the harms and benefits of machine learning systems, correlated to what has been called “the responsibility-gap” (Matthias 2004). According to Matthias, the increase in autonomy of AI systems based on machine learning technology (“learning automata”) leads to situations where we face a void or gap in ascriptions of responsibility. On the one hand, if self-learning AI systems are initially programmed by humans, it looks as if they cannot be moral agents and thus cannot bear blame for the consequences of their actions and decisions. On the other hand, if AI systems are such good self-learners that human programmers cannot foresee all possible consequences of their actions, then humans cannot bear blame for AI actions or decisions, either.

The theoretical and practical existence of responsibility gaps have been challenged or even denied by some (Hindriks and Veluwenkamp 2023; Himmelreich 2019; Königs 2022; List 2021; Tigard 2021). For example, Tigard (2021) emphasizes that it might not even be relevant to bridge the responsibility gap opened by AI systems (a “techno-responsibility gap”), as there is nothing to bridge once we conceptually clarify the topic. Alternatively, AI systems might be appropriate responsibility *loci* themselves, just like group agents such as organizations (List 2021), which, again, dissolves the gap. Furthermore, it might be the case that not all responsibility gaps need to be bridged (Munch et al. 2023), for instance not in cases where tragic choice is involved (Danaher 2022).

Others take responsibility gaps seriously and try to bridge them. For instance, Marino and Tamburini (2006: 50) suggest developing “responsibility ascriptions policies” that delineate the responsibility of individual engineers and computer scientists as well as their organizations. Putting humans back in the loop and making explicit the reasons that ground decisions provided by AI systems can potentially close the gap (Baum et al. 2022). Another direction is to distribute responsibility among the multiple actors that have a causal contribution to the effects generated by AI (Taddeo and Floridi 2018), with the alternative of ascribing vicarious moral responsibility (Pascucci and Glavaničová, 2022). A model of collaborative agency is developed by Nyholm (2018) to argue that humans can be held responsible for what learning automata do, in a principal–agent relationship fashion.

A more promising line of dealing with responsibility gaps seems to be that of nuancing the problem and emphasizing that there is no single, but rather multiple types of responsibility gaps. Santoni de Sio and Mecacci (2021) identify four distinct responsibility gaps: culpability, moral accountability, public accountability, and active/forward-looking responsibility. Other types of responsibility gaps that were identified refer, for instance, to retribution gaps (Danaher 2016) and vulnerability gaps (Vallor and Vierkant 2024).

### AI avatars, epistemic gaps, and the proxy problem

In a recent article, Paula Sweeney (2023) has made the case that highly autonomous AI systems in the form of “Autonomous Advanced Avatars” open one more type of responsibility gaps, namely epistemic gaps. She defines this form of AI avatars as one that “could dynamically represent a living person who is not in the environment that the system is operating in” (Sweeney 2023: 530). Drawing on Luciano Floridi’s (2015) analysis of proxy relationships, Sweeney highlights that humans and avatars do not stand in a perfect identity relationship but are instead in a “proxy relationship”. Acting by proxy is possible “because something both represents and replaces something else” (Sweeney 2023: 527). Avatars as proxies both “stand for a person” and “stand in for a person”, which positions avatars as “perfect proxies” of their human users.

Importantly, “the proxy relation is one in which the represented person can be held responsible for the actions of their proxy” (Sweeney 2023: 526), hence the epistemic gap. Even though, ideally, the proxy acts or decides in perfect match with the action or decision of the person represented, in practice, however, proxies fall short of this perfect match given the contextual nuances of the decision-making process (Sweeney 2023). This results in the outcome that “the represented individual will directly and personally bear the consequences of mishaps and the responsibility for any resulting harm to others”, “despite the epistemic gap that will exist between the agent and their proxy” (Sweeney 2023: 536).

While I agree with Sweeney (2023) that AI Avatars are (or can be) full proxies and this proxy relationship is problematic in terms of opening (undesirable) epistemic gaps, I would like to take a step further. In the next sections, I argue that the use of GenAI avatars is problematic in several other (related) ways, by (a) introducing a proxy-control paradox and (b) opening a related control gap, which, together with the epistemic gap identified by Sweeney, (c) leads to a new responsibility gap, which I will call the “proxy gap”.

### GenAI avatars

In discussing proxy gaps opened by AI avatars, I argue that these gaps appear even in apparently less complicated use cases of semi-autonomous avatars, where the human partly teleoperates their avatar—instead of being completely out-of-the-loop, as in use cases of other highly autonomous digital artificial replicas referred to as “digital human twins” (Lin et al. 2024), “digital duplicates” (Danaher and Nyholm 2024), or “digital doppelgängers” (Iglesias et al 2024). In the remaining of the article, I particularly focus on personal and personalized semi-autonomous generative AI avatars, or, simply, GenAI avatars, with applications in digital environments and social robotics.

I take GenAI avatars to cover three main characteristics: a) personal avatars in the form of digital or robotic representations of real human persons that are b) personalized through LLM training on data pertaining to particular individuals, to complement, enhance or even replace the reasoning and acting of the person being replicated, and c) cover varying degrees of autonomy and human control and are best described as semi-autonomous, because they are neither fully controlled by the replicated individual, nor are they fully autonomous (they alternate real-time human operation with no human oversight).

Relevant examples of digital GenAI avatars include lab experiments by Kawahara et al. (2025:73–74) designing “a hybrid of an autonomous dialogue system and a human-operated avatar”, with applications in customer care, where the avatar can be a “virtual agent” and the human teleoperator would be “expected to be able to serve three or more users simultaneously”, given that “the majority of dialogue can be handled in an autonomous manner such as explanation or listening”. On the side of robotic GenAI avatars, cybernetic avatars such as Geminoid HI-6 and the Moonshot avatar-symbiotic society (Ishiguro et al. 2025), as well as human avatar systems such as ICub (Dafarra et al. 2024), include robotic avatars that combine LLMs with varying levels of human teleoperation. Envisioned use of semi-autonomous AI avatars is put forward by one of the finalist teams in the ANA Avatar XPrize Competition, with Park et al. (2024) reporting “shared autonomy control” as a possible solution to remote operation difficulties that most teleoperated robotic avatars faced when they had to use a screw as part of the competition. This solution integrates “both manual control by a human and autonomous control by a robot”, whereby “the operator’s remote manual control moves the robot’s position or approaches the target object”, while the “robot’s autonomous control aligns its hand with the object or adjusts the position and direction of the held tool to match that of the target object” (Park et al. 2024).

## The proxy-control paradox

Because they rely on the LLMs technology, GenAI avatars complicate the representation relationship between the avatar and the human user, which is now shaped by the generative AI technology. Prior use of avatars in lack of such technologies displayed a rather direct and explicit link between the user, the avatar, and the outcome, whereby the user would persistently and significantly control the output of their avatar. See, for instance, digital avatars in Second-Life or robotic avatars such as Geminoid HI-1, that did not rely on AI technologies and where the user was in direct control and could reasonably predict the outcome of their avatar. However, as I will argue next, the use of GenAI avatars disrupts this agentic control between human users and avatar outcomes, even when a human operates their LLM avatar in real time. This disruption of agentic connection is grounded in what I label as a “proxy-control paradox”.

The proxy-control paradox can be seen as a subset of the broader idea of the control paradox whereby “in order to increase (or improve) control, we must cede it” (di Nucci 2020: xiv). In his in-depth analysis of the notion of control paradox and some of its applications, di Nucci (2020) emphasizes that technological innovation generally involves a form of increased control by ceding some (or more) control to the very technology that is introduced to obtain improved control. Nonetheless, the control paradox is not limited to complex technologies like AI systems but covers various human practices that involve a form of delegation, including politics, where “trying to gain more and better control simultaneously increases the risk of losing control” (di Nucci, 2020: xiv).

Use of GenAI avatars involves a trade-off of human agency in terms of control. On the one hand, using avatars, we aim to gain enhanced human capabilities in the digital or physical world. We achieve a form of presence in places or contexts that we normally cannot access, such as a digital outer space or a physically toxic environment. Computational and robotics technologies enhance not only our physical, but also our cognitive abilities. For instance, current or near-future semi-autonomous GenAI avatars that have a human partly in the loop can automatically adjust our accent in a foreign language or translate our speech in real-time in an online lecture; the generative AI technology powering an avatar can visually prompt real-time advice over the dangers in the surrounding environment where the avatar is placed, enabling the individual represented by the avatar to make better decisions in conflict negotiation, for instance. Near-future GenAI avatars could make sense of the movement of the other participants in the interaction and react instantly by literally dodging a bullet, even when the human user does not have the time to input this command. However, for digital or physical avatars to expand human capabilities, or replace humans in some contexts, we need to use a form of generative AI to bridge the distance between the human represented and the output of their avatar. This results in transferring significant human control to technology and losing of the direct agentic connection with the outcome of our actions.

GenAI avatars, therefore, involve a “proxy-control paradox”: in trying to achieve improved control over the desired outcome of their avatar, humans need to delegate control over the process leading to that outcome to the GenAI technology. An important distinction here is between *process control* and *outcome control*. In the case of semi-autonomous GenAI avatars, human users are enabled to achieve more outcome control over their avatars only provided they give up process control in favor of the generative AI technology. This results in a more distant and diffuse connection to the outcome of what humans do via their avatars. In lack of the generative AI technology, the users would be in direct control of their avatar—there would, of course, be cases of glitches, malfunctions, but not a genuine transfer of control. With use of GenAI, the individual loses direct agentic control over their avatars, which is transferred to the generative AI technology that models the representation relationship between the individual and the avatars.

Ideally, the proxy relationship involves a perfect representation between individuals and avatars, with successful delegation of control from the former to the latter. This type of successful delegation would not result in a loss of control but would amount to “not having to do any direct controlling while remaining in control” (di Nucci, 2020: xxv). However, given limits of control delegation and the indirect agentic connection resulting from lack of process control, the proxy-control paradox surrounding the use of semi-autonomous GenAI avatars offers substantive reasons to doubt the responsibility of the human user for what is done through their avatar.

Lack of control in GenAI avatars reiterates responsibility gaps opened highly autonomous AI systems or social robots coupled with AI, where humans are out-of-the-loop and we have difficulty in placing responsibility with designers, programmers, users, manufacturers, and so forth. Unlike more general uses of AI systems, use of GenAI avatars rests on a proxy representation relationship, suggesting that we have a somewhat predetermined moral responsibility of the human user of the avatar, as in the case of teleoperated avatars without AI technologies. However, in the case of semi-autonomous GenAI avatars, this direct connection is counterbalanced by use of LLMs technologies, which actively shapes the relationship between the represented individual and their avatar. Given that individuals represented by semi-autonomous GenAI avatars have no process control over the relevant actions performed by their avatars, we cannot hold them morally blameworthy for the outcome of their avatars—at least not in the same straightforward way as we would have done in lack of the generative AI technology shaping the representation relationship between individuals and their avatars.

## Mind the proxy gap!

GenAI avatars bring further complications and nuances to human–avatar proxy relationship. GenAI avatars as representations of real individuals function not merely as passive proxies but rather as operational intermediaries with significant agency, more than precise extensions of user intent. Use of GenAI avatars, therefore, opens “proxy gaps”: moral responsibility gaps that refer to the representation relationship between the avatar and the human user, shaped by the generative AI technology. The human–avatar “proxy arrangement” (Sweeney 2023) comes with the expectation of a good-enough, if not perfect, match between the one represented and the other representing them, which means that the proxy both takes the place of and acts on behalf of the original. GenAI avatars offer the promise of being such perfect proxies for individuals, but something is misleading about this arrangement.

GenAI avatars require us to lose some level of agency in terms of control and knowledge, which are precisely the two main criteria widely used in general moral philosophy and the applied field of AI ethics to ascribe moral responsibility (Coeckelbergh 2020; Fischer & Ravizza 1993; Hakli & Mäkelä, 2019; Sison & Redín, 2021). On the one hand, the freedom or control condition requires that an agent has relevant control over the circumstances of their action or can choose freely among alternative possibilities of action (Fischer & Ravizza 1993), to be ascribed moral responsibility. On the other hand, the epistemic or knowledge condition requires that agents possess relevant information and the capacity to deliberate about the circumstances and implications of their actions (Clarke 1992; McKenna and Widerker, 2003) to be adequate targets for ascriptions of moral responsibility.

How are the two criteria for moral responsibility impaired using GenAI avatars? First, the control condition cannot be fully met by the human represented by the avatar given the intervention of the GenAI technology, which automatically adjusts, e.g., the actions, words, or postures of the avatar in the digital or physical environment. While the GenAI technology empowers the human teleoperator with regards to the avatar output, for instance by enabling fine-grained gestures otherwise not possible, such as touching a butterfly, it also takes over the direct control of the human teleoperator. Freedom of action is subsequently impaired, as the human represented by the avatar cannot choose in real time the way their avatar powered by GenAI will translate their words into a foreign language, for example.

Drawing on key elements of control and technology highlighted by di Nucci (2020) and Nyholm (2022), the following three main aspects of control can be useful for articulating the proxy-control gap more clearly: (1) control can be direct or indirect; (2) control can be limited yet meaningful; (3) control is multi-dimensional and can be split across multiple agents. As Vacek (2024) highlights, having complete control over AI systems is practically impossible, given the very reason for designing AI systems in the first place; instead, limited yet considerable control is both achievable and desirable. Furthermore, the idea of “meaningful human control” over autonomous systems was introduced by Santoni de Sio and van den Hoven (2018) to describe relevant control over AI systems. While meaningful human control over AI systems does not necessarily require full or direct control, it requires that humans meet the “tracing” and “tracking” requirements, which ensure the necessary threshold for ascribing moral responsibility to human agents for the outcome of AI systems.

Use of GenAI avatars can potentially undermine both the tracking and the tracing conditions required for meaningful human control for the outcomes of AI systems in general and of GenAI avatars in particular. First, the “tracking” condition refers to the requirement that an AI system responds to relevant moral reasons of their human designers and deployers, as well as to relevant cues in the environment in which the system operates. However, the LLM technology shapes the actions leading to the outcome of the GenAI avatar to the extent that these actions will not respond to reasons of the human user and will instead be adjusted according to the training data sets used by the model. Second, the “tracing” condition requires that the system grants the possibility to correlate its outcomes with at least one human person along the chain of design and operation. Here, again, the LLM technology shapes the actions leading to the outcome of the GenAI avatar to the extent that these actions will not correlate all the times with the input from the human using the avatar and instead will automatically adjust relative to the environment in which the GenAI avatar operates or is operated.

Second, the epistemic condition cannot be fully met by the human represented by an avatar because the GenAI technology processes contextual information over the physical or digital environment and turns this contextual input into action output in real time. This turns the human user into a passive witness of their avatar output. Besides situations where individuals prompt the avatar to look into a certain direction and collect particular pieces of information over the environment, there will be cases where humans do not have time to intervene in-between the processing of contextual information and action. In fact, this situation is part of the very process of acting via GenAI avatars, because AI algorithms are actually supposed to process details in real time and accordingly. This becomes apparent in time-sensitive situations, such as emergency rescue operations, when the ability to process information and act decisively becomes paramount. In such cases, we need to delegate to the GenAI technology the capacity to correlate real-time-input with instant action output.

To some extent, this back-and-forth move between avatars and human users resembles the way pilots engage with their airplanes. For instance, in case of low visibility, as well as in takeoff and landing, most control of the plane is delegated to the autopilot system, which is better equipped to handle such complicated situations than human pilots. However, pilots are not automatically ascribed responsibility in case of an accident, and each time an investigation is needed to determine whose fault the crash was. Human error covers cases when, for instance, pilots did not interpret well and did not properly react to the technical signals they received, when they were supposed to do so. However, they do not bear blame for cases when the autopilot either malfunctioned or, despite functioning well, led to a crash. When human pilots delegate process control to the plane autopilot to gain more outcome control, they do not bear moral responsibility for cases when less outcome control is achieved, and accidents happen. At most, human pilots might bear responsibility for delegating control to the autopilot when they should not have, for instance when they know that the autopilot errs in a specific context and human direct control is preferable. Unlike the airplane case, GenAI avatars are proxies for their human users and, in addition, are personalized to the data of the individual they are representing. This proxy representation relationship leads to the high probability that humans behind avatars are more easily and straightforwardly ascribed moral responsibility for situations when their avatars are in control, without even considering an investigation as in cases of airplane crashes.

This probability is illustrated by studies showing that humans tend to see avatar outcomes as their own, even when there is lack of evidence of a causal relationship between what the human user does and the output of their avatar. For instance, in a study conducted by Aymerich-Franch, Kishore, and Slater (2020), participants felt guilt during a form of robot avatar embodiment where they experienced the body of a humanoid robot as if it were their own. As the study shows, when the robot avatar spontaneously verbally abused someone during a conversation using the participant’s voice, without there being any form of input from the human teleoperator, humans felt the need to apologize to their interlocutor for the way their robot avatar misbehaved. Given the direct personal connection established between the individual and their semi-autonomous GenAI avatar, we can reasonably expect that moral responsibility will straightforwardly be ascribed to the individual for the output of their avatar, even when the human does not meet the relevant control criterion for moral responsibility.

The use of digital and robotic GenAI avatars, therefore, opens new responsibility gaps, namely proxy gaps, which refer to the exact nature of the relationship between the human users and their avatars, shaped by generative AI. Nonetheless, there seem to exist situations when we can rightfully consider the individual represented by the avatar to be morally responsible for the outcome of their GenAI avatars. I explore in the next section possible criteria that ground individual moral responsibility for GenAI outcomes.

## When are individuals morally responsible for the outcomes of their GenAI avatars?

Justified ascriptions of moral responsibility to a person need to consider the capacity of that person to cause and choose what they want, to have relevant control over what they do, as well as to be knowledgeable of the context of their actions and understand the implications of these actions (Constantinescu et al. 2022). To avoid ascribing ungrounded moral responsibility and, subsequently, unjustifiably blame or praise the human user of semi-autonomous GenAI avatars, we need to refer to a set of minimally relevant criteria. I delineate and explain below a minimal set of four criteria that need to be met to rightfully consider the individual represented by the avatar to be morally responsible for the outcome of their GenAI avatars and thus plug, in specific situations, proxy-responsibility gaps. The first two criteria ensure that a relevant threshold for the epistemic condition for moral responsibility ascriptions is met by the human user, while the last two criteria ensure the threshold for the control condition. This leaves open the possibility to add new criteria, once we estimate that moral responsibility is inadequately placed with the human users.

### Understanding

First, and related to the epistemic condition for moral responsibility, the human user must correctly understand the fact that the digital or robotic avatar they are using relies on multimodal generative AI technology and that this technology will play an active part in shaping the relationship between the human and the output of their avatar. The “understanding” condition resembles to some extent with informed consent used in bioethics research and the consent condition recently put forward by Danaher and Nyholm (2024) as part of their minimally viable permissibility principle (MVPP) for the creation and use of digital duplicates. In the case of semi-autonomous GenAI avatars discussed here, the understanding condition means that the human user understands—and agrees—that the GenAI technology will not only complement, but often will take over their actions, postures, and uttering in real time; moreover, and more importantly, that this will ensure a higher control over the intended outcome of their action via avatars, while, at the same time, a lower control over what the avatar actually does in the process of achieving the desired outcome. Nonetheless, even when this criterion is met, prior issues related to informed consent in bioethics arise anew, such as the range of future actions for which consent is offered, given the difficulties to ensure that the user was provided enough information to fully understand the way the GenAI avatar functions.

### Personalization

Second, and related to the epistemic condition for moral responsibility, training of the generative AI technology must rely on personal data of the individual user and, when larger training data sets are needed to achieve relevant accuracy, these data sets need to come from exemplar behavior in fairly similar contexts of action or reasoning. The AI algorithm needs to be fed with the values, principles or goals of the individual who is using the semi-autonomous GenAI avatar, if we are to call it a personal or personalized avatar. When larger data sets are needed, these must be relevantly aligned with personal data of the individual user. One cannot be held morally responsible for the outcomes of their GenAI avatar if the training data sets are not fully personalized but only generic. In addition, to be proper bearers of moral responsibility, users would need to be able to opt for one GenAI technology over the other, considering a multitude of factors including efficiency but also security, reliability or ethical issues. In use cases covering industrial deployment and institutionalized settings where human users would have little power to choose between more and less performant GenAI technology, resulting in less efficient personalization, moral responsibility cannot be placed on the individual. Instead, we need to either place blame with the organization deploying the avatar system or consider this to be a genuine responsibility void.

### Veto

Third, and related to the control condition for moral responsibility, the human user should have the power to prevent an action of the avatar along the process of using their avatar, which best translates as a right to veto. Even though best outcome control can only be achieved by delegating process control to the generative AI technology powering one’s avatar, this delegation should not be understood in absolute terms. Moral responsibility can be ascribed to the human user only provided they have the possibility to intervene when they disagree with the actions taken by their GenAI avatar towards achieving the desired goals. In lack of this criterion, the human user would not exert indirect agency relative to the outcome of their GenAI avatar, but rather diminished agency, which is not a good-enough ground to hold them morally responsible. Diminished agency would lead to diminished responsibility of the human user. The choices the GenAI technology makes for the individual represented need to be the choices of the individual and not the other way around. This does not necessarily mean that for each and every action of the avatar, the human needs to exert direct control, given the complex granularity of sub-actions, but rather that at some initial point of use and at specific steps in the process, the individual is in the position to decide the extent to which they delegate agency to the GenAI avatar. This requires a form of continuous and constant feedback from the generative AI technology to the individual and back. The key point of this condition is to avoid burdening the human user with full moral responsibility for what they do via their GenAI avatar, in cases where there is not enough correspondence between human decision and avatar action.

### Outcome

Fourth, and related to the control condition for moral responsibility, the design of the GenAI avatar must in fact enable the human user to gain more outcome control over their avatars. The proxy-control paradox provides a rationale to ascribe moral responsibility to the human user, even in lack of process control, based on the following reasoning: if more outcome control of the human user is achieved using generative AI technology, then it follows that responsibility for the outcome stands with the human user. However, this is dependent upon potential malfunctions of the avatar system, as well as on the correct description of potential risks by the organization that develops or owns the GenAI avatar. If the generative AI technology errs in what is supposed to do, for instance by incorrect visual interpretation of the physical environment or by wrong translation of the words uttered by the human controller, then we cannot ascribe moral responsibility to the human. Likewise, if the organization developing, manufacturing, or maintaining the avatar system does not explicitly mention risks of use, similar to a medicine leaflet or a safety manual for technical equipment, we cannot straightforwardly ascribe moral responsibility to the human represented by the avatar. In such cases, we need to either place blame with the organization deploying the avatar system, who sold their product or service as fully reliable, when it was not, or consider this to be a genuine responsibility void.

An interesting scenario regarding the outcome criterion concerns cases where the GenAI avatar operates as it is supposed to, without malfunctions, but it still leads to undesirable outcomes. For instance, when GenAI avatars provide real-time translation in virtual diplomatic settings, even a perfect technical translation may fail to capture crucial cultural nuances and contextual cues. In diplomatic translation, interpreting context often matters more than literal translation, requiring complex skills that AI may not fully replicate. This can lead to miscommunication even when the AI functions as designed. One relevant real-case situation involving human diplomatic translation is described by Rosado (2018): at the 1987 White House summit, Reagan welcomed Gorbachev by noting they were meeting as “adversaries” rather than allies. U.S. interpreter Zarechnak faced a challenge translating this word, as its Russian equivalent “protivniki” had negative connotations similar to “disgusting”. He opted for the Russian translation for “competitors” instead, which was positively received by Gorbachev.

When individuals who use semi-autonomous GenAI avatars do not meet the four proposed criteria to be ascribed moral responsibility for the outcomes of their avatars, other agents might be relevant bearers of moral responsibility, including collective agents such as institutions deploying or developing GenAI avatars. It is beyond the scope of the current article to provide a developed account for possible ways to bridge proxy gaps by means of finding other individual or collective agents who might bear moral responsibility for the outcomes of personal GenAI avatars. Nonetheless, it is important to stress that proxy gaps remain genuine responsibility gaps in cases where no agent is a fitted candidate to meet the relevant criteria for moral responsibility ascriptions for the outcomes of GenAI avatars.

## Conclusion and implications

In this article, I emphasized that a recent development of digital and robotic avatars, that represent individual human persons, has the potential to open a new type of responsibility gaps, that adds to those already opened by increasingly autonomous AI systems. Namely, I argued that GenAI avatars open proxy gaps: situations when, given the representation relationship between the human user and their GenAI avatar, shaped by the nature of the multimodal AI technology, we have no one to blame for the outcome of GenAI avatars. This happens because the most obvious potential bearer of moral responsibility, namely the human user of the avatar, cannot meet the threshold criteria for the epistemic and control requirements that ground ascriptions of moral responsibility. In addition to the epistemic gap previously introduced by Sweeney (2023) in relation to autonomous advanced avatars, I made the point that GenAI avatars also open a control gap rooted in the proxy-control paradox, which consists in the necessity to delegate significant control to the generative AI technology, to gain more control over the outcome of GenAI avatars. Taken together, the epistemic gap and the control gap amount to a novel responsibility gap—the proxy gap introduced by GenAI avatars.

Furthermore, I suggested that we can still rightfully ascribe moral responsibility to the individual user of GenAI avatars, provided the following minimal set of criteria is met: (1) understanding the limits and potential of the GenAI avatar, (2) personalization of training data sets to correspond to user identity and preferences, (3) having the possibility to veto the outcome of GenAI avatars and, finally, (4) achieving more outcome control than the user would have achieved in lack of the multimodal LLMs technology. When such criteria are not met, humans might be ascribed moral responsibility for something that their GenAI avatar does out of their direct control and knowledge.

A relevant implication concerns the level of autonomy for personal avatars that is advisable for the future. Given the proxy-responsibility gaps opened by GenAI avatars that partly have a human in loop, there are important concerns over future use of personal AI avatars that potentially display full autonomy and operate independently from a human user. Beyond enforcing relevant safeguards in the development, deployment, and use of highly autonomous AI avatars, one relevant regulatory measure would be to encourage the use of personal AI avatars with lower level autonomy, in the detriment of fully autonomous avatars. In use cases covering institutional deployment of GenAI avatars, organizations would need to meet specific requirements in terms of avatar use and explicitly mention how they would be held responsible for the outcome of avatars tailored to use of individual members, how they would ensure that criteria such as those delineated in this article are met, alongside field-specific additional requirements particularized, for instance, to healthcare, education, military. Despite these avatars representing real people, we do not have the relevant normative grounds to blame the humans they are representing for the outcomes of these avatars, when threshold criteria for control or knowledge are not met. This leaves us with the undesirable outcome of dealing with moral and legal responsibility voids.

## Data Availability

No datasets were generated or analyzed during the current study.
